# 2,6-Bis(4-methoxy­benzyl­idene)cyclo­hexa­none

**DOI:** 10.1107/S160053680901112X

**Published:** 2009-03-31

**Authors:** Deyun Liu, Guohua Chen

**Affiliations:** a Liaocheng Vocational and Technical College, Liaocheng 252059, People’s Republic of China

## Abstract

In the title mol­ecule, C_22_H_22_O_3_, the central cyclo­hexa­none ring adopts an envelope conformation. The two outer aromatic rings form a dihedral angle of 19.3 (2)°. The crystal packing exhibits weak inter­molecular C—H⋯O hydrogen bonds.

## Related literature

For background, see: Tanaka *et al.* (2000 ▶). For a related structure, see: Brinda, Mudakavi *et al.* (2007 ▶).
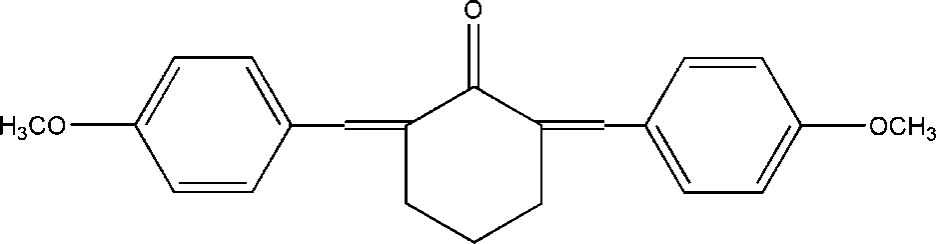


## Experimental

### 

#### Crystal data

C_22_H_22_O_3_
*M* *_r_* = 334.40
Monoclinic, 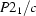

*a* = 9.0129 (8) Å
*b* = 9.4874 (10) Å
*c* = 20.9416 (17) Å
β = 100.518 (1)°
*V* = 1760.6 (3) Å^3^
*Z* = 4
Mo *K*α radiation
μ = 0.08 mm^−1^
*T* = 298 K
0.45 × 0.17 × 0.15 mm

#### Data collection

Bruker SMART APEX CCD area-detector diffractometer
Absorption correction: multi-scan (*SADABS*; Sheldrick, 1996 ▶) *T*_min_ = 0.964, *T*_max_ = 0.988
9092 measured reflections
3105 independent reflections
1233 reflections with *I* > 2σ(*I*)
*R*_int_ = 0.065

#### Refinement

*R*[*F* ^2^ > 2σ(*F* ^2^)] = 0.061
*wR*(*F* ^2^) = 0.170
*S* = 0.87
3105 reflections
228 parameters
H-atom parameters constrained
Δρ_max_ = 0.26 e Å^−3^
Δρ_min_ = −0.12 e Å^−3^

### 

Data collection: *SMART* (Siemens, 1996 ▶); cell refinement: *SAINT* (Siemens, 1996 ▶); data reduction: *SAINT*; program(s) used to solve structure: *SHELXS97* (Sheldrick, 2008 ▶); program(s) used to refine structure: *SHELXL97* (Sheldrick, 2008 ▶); molecular graphics: *SHELXTL* (Sheldrick, 2008 ▶); software used to prepare material for publication: *SHELXTL*.

## Supplementary Material

Crystal structure: contains datablocks I, global. DOI: 10.1107/S160053680901112X/cv2532sup1.cif

Structure factors: contains datablocks I. DOI: 10.1107/S160053680901112X/cv2532Isup2.hkl

Additional supplementary materials:  crystallographic information; 3D view; checkCIF report

## Figures and Tables

**Table 1:** Hydrogen-bond geometry (Å, °)

*D*—H⋯*A*	*D*—H	H⋯*A*	*D*⋯*A*	*D*—H⋯*A*
C14—H14*B*⋯O2^i^	0.96	2.67	3.512 (5)	146
C4—H4*A*⋯O1^ii^	0.97	2.61	3.510 (5)	154

Symmetry codes: (i) 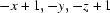; (ii) 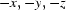.

**Fig. 1.**
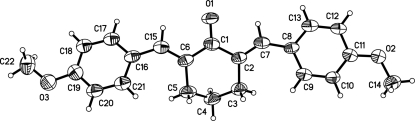
View of (I) showing the atomic numbering scheme and 40% probability displacement ellipsoids.

## supplementary crystallographic information

### Comment

The use of organic syntheses without volatile, often flammable, expensive and toxic solvents strongly reduces the waste production and many fundamental processes have proven to be achievable through efficient procedures characterized by high simplicity of set-up and work-up (Tanaka *et al.*, 2000). In this paper, we describe a solvent-free protocol used in the synthesis of the title compound, (I), starting from the fragrant aldehydes and cyclohexanone in the presence of NaOH.

In (I) (Fig. 1), the bond lengths and angles are normal and correspond to those observed in 4-methyl-2,6-bis(2-naphthylmethylene) cyclohexan-1-one (Brinda, Mudakavi *et al.*, 2007). The central cyclohexanone ring adopts an envelope conformation. The mean planes of two rings - C8—C13 and C16—C21 - form a dihedral angle of 19.3 (2)°. The crystal packing exhibits weak intermolecular C—H···O hydrogen bonds (Table 1).

### Experimental

2-Methoxylbenzaldehyde (4 mmol) and cyclohexanone (2.0 mmol), NaOH (4.0 mmol) were mixed in 50 ml flash under sovlent-free condtions After stirring for 15 min at 293 K, tthe resulting mixture was washed with water for several times for removing NaOH, and recrystalized from ethanol, and afforded the title compound as a crystalline solid. Elemental analysis: calculated for C_22_H_22_O_3_: C 79.02, H 6.63%; found: C 69.93, H 6.65%.

### Refinement

All H atoms were positioned geometrically (C—H = 0.93–0.97 Å) and refined using a riding model, with *U*_iso_(H) = 1.2–1.5*U*_eq_(C).

### Crystal data

**Table:**

C_22_H_22_O_3_	*F*(000) = 712
*M**_r_* = 334.40	*D*_x_ = 1.262 Mg m^−^^3^
Monoclinic, *P*2_1_/*c*	Mo *K*α radiation, λ = 0.71073 Å
*a* = 9.0129 (8) Å	Cell parameters from 935 reflections
*b* = 9.4874 (10) Å	θ = 2.4–25.2°
*c* = 20.9416 (17) Å	µ = 0.08 mm^−^^1^
β = 100.518 (1)°	*T* = 298 K
*V* = 1760.6 (3) Å^3^	Prism, yellow
*Z* = 4	0.45 × 0.17 × 0.15 mm

### Data collection

**Table:**

Bruker SMART APEX CCD area-detector diffractometer	3105 independent reflections
Radiation source: fine-focus sealed tube	1233 reflections with *I* > 2σ(*I*)
graphite	*R*_int_ = 0.065
φ and ω scans	θ_max_ = 25.0°, θ_min_ = 2.0°
Absorption correction: multi-scan (*SADABS*; Sheldrick, 1996)	*h* = −10→10
*T*_min_ = 0.964, *T*_max_ = 0.988	*k* = −11→11
9092 measured reflections	*l* = −23→24

### Refinement

**Table:**

Refinement on *F*^2^	Primary atom site location: structure-invariant direct methods
Least-squares matrix: full	Secondary atom site location: difference Fourier map
*R*[*F*^2^ > 2σ(*F*^2^)] = 0.061	Hydrogen site location: inferred from neighbouring sites
*wR*(*F*^2^) = 0.170	H-atom parameters constrained
*S* = 0.87	*w* = 1/[σ^2^(*F*_o_^2^) + (0.076*P*)^2^] where *P* = (*F*_o_^2^ + 2*F*_c_^2^)/3
3105 reflections	(Δ/σ)_max_ < 0.001
228 parameters	Δρ_max_ = 0.26 e Å^−^^3^
0 restraints	Δρ_min_ = −0.12 e Å^−^^3^

### Special details

**Table:**

Geometry. All e.s.d.'s (except the e.s.d. in the dihedral angle between two l.s. planes) are estimated using the full covariance matrix. The cell e.s.d.'s are taken into account individually in the estimation of e.s.d.'s in distances, angles and torsion angles; correlations between e.s.d.'s in cell parameters are only used when they are defined by crystal symmetry. An approximate (isotropic) treatment of cell e.s.d.'s is used for estimating e.s.d.'s involving l.s. planes.
Refinement. Refinement of *F*^2^ against ALL reflections. The weighted *R*-factor *wR* and goodness of fit *S* are based on *F*^2^, conventional *R*-factors *R* are based on *F*, with *F* set to zero for negative *F*^2^. The threshold expression of *F*^2^ > σ(*F*^2^) is used only for calculating *R*-factors(gt) *etc*. and is not relevant to the choice of reflections for refinement. *R*-factors based on *F*^2^ are statistically about twice as large as those based on *F*, and *R*- factors based on ALL data will be even larger.

### Fractional atomic coordinates and isotropic or equivalent isotropic displacement parameters (Å^2^)

**Table:**

	*x*	*y*	*z*	*U*_iso_*/*U*_eq_	
O1	0.1376 (3)	−0.1927 (3)	0.02703 (11)	0.1012 (9)	
O2	0.4818 (3)	−0.0038 (2)	0.41111 (13)	0.0905 (8)	
O3	0.0867 (3)	0.0536 (3)	−0.35644 (17)	0.1062 (9)	
C1	0.1981 (4)	−0.0760 (4)	0.02660 (19)	0.0782 (10)	
C2	0.2658 (4)	−0.0065 (3)	0.0884 (2)	0.0729 (10)	
C3	0.3239 (4)	0.1430 (3)	0.08487 (18)	0.0941 (12)	
H3A	0.3018	0.1963	0.1215	0.113*	
H3B	0.4327	0.1404	0.0883	0.113*	
C4	0.2551 (5)	0.2176 (4)	0.02279 (19)	0.1055 (14)	
H4A	0.1479	0.2305	0.0217	0.127*	
H4B	0.3007	0.3101	0.0220	0.127*	
C5	0.2779 (5)	0.1360 (4)	−0.03590 (18)	0.0973 (12)	
H5A	0.3851	0.1263	−0.0359	0.117*	
H5B	0.2331	0.1869	−0.0748	0.117*	
C6	0.2063 (4)	−0.0095 (3)	−0.0364 (2)	0.0754 (10)	
C7	0.2725 (4)	−0.0772 (3)	0.1430 (2)	0.0744 (10)	
H7	0.2294	−0.1662	0.1361	0.089*	
C8	0.3302 (4)	−0.0512 (3)	0.21109 (18)	0.0655 (9)	
C9	0.4222 (4)	0.0636 (4)	0.2354 (2)	0.0812 (10)	
H9	0.4495	0.1291	0.2066	0.097*	
C10	0.4728 (4)	0.0810 (3)	0.30125 (19)	0.0760 (10)	
H10	0.5326	0.1585	0.3158	0.091*	
C11	0.4371 (4)	−0.0127 (4)	0.3454 (2)	0.0746 (10)	
C12	0.3475 (4)	−0.1281 (3)	0.3233 (2)	0.0753 (10)	
H12	0.3210	−0.1932	0.3525	0.090*	
C13	0.2984 (4)	−0.1449 (3)	0.2575 (2)	0.0789 (10)	
H13	0.2403	−0.2238	0.2434	0.095*	
C14	0.5918 (5)	0.0992 (4)	0.43567 (18)	0.1055 (13)	
H14A	0.6829	0.0810	0.4194	0.158*	
H14B	0.6125	0.0951	0.4823	0.158*	
H14C	0.5545	0.1911	0.4220	0.158*	
C15	0.1460 (4)	−0.0755 (3)	−0.0911 (2)	0.0747 (10)	
H15	0.1021	−0.1614	−0.0840	0.090*	
C16	0.1343 (4)	−0.0418 (3)	−0.1593 (2)	0.0709 (10)	
C17	0.0221 (4)	−0.1023 (3)	−0.2053 (2)	0.0777 (10)	
H17	−0.0428	−0.1659	−0.1907	0.093*	
C18	−0.0001 (4)	−0.0757 (4)	−0.2704 (2)	0.0792 (10)	
H18	−0.0786	−0.1184	−0.2988	0.095*	
C19	0.0973 (5)	0.0166 (4)	−0.2928 (2)	0.0862 (12)	
C20	0.2143 (5)	0.0738 (4)	−0.2494 (2)	0.0884 (12)	
H20	0.2823	0.1329	−0.2646	0.106*	
C21	0.2335 (4)	0.0466 (4)	−0.1850 (2)	0.0894 (11)	
H21	0.3142	0.0874	−0.1572	0.107*	
C22	−0.0240 (5)	−0.0063 (4)	−0.4044 (2)	0.1223 (16)	
H22A	−0.0036	−0.1049	−0.4082	0.184*	
H22B	−0.0231	0.0395	−0.4452	0.184*	
H22C	−0.1213	0.0054	−0.3925	0.184*	

### Atomic displacement parameters (Å^2^)

**Table:**

	*U*^11^	*U*^22^	*U*^33^	*U*^12^	*U*^13^	*U*^23^
O1	0.117 (2)	0.0614 (16)	0.120 (2)	−0.0281 (15)	0.0088 (15)	0.0043 (14)
O2	0.097 (2)	0.0870 (18)	0.087 (2)	−0.0124 (15)	0.0157 (15)	0.0058 (14)
O3	0.128 (3)	0.090 (2)	0.104 (2)	0.0029 (17)	0.0314 (19)	−0.0034 (17)
C1	0.072 (3)	0.059 (2)	0.105 (3)	0.0060 (19)	0.019 (2)	0.008 (2)
C2	0.066 (2)	0.053 (2)	0.098 (3)	−0.0002 (17)	0.012 (2)	−0.004 (2)
C3	0.107 (3)	0.061 (2)	0.109 (3)	−0.011 (2)	0.005 (2)	0.003 (2)
C4	0.133 (4)	0.053 (2)	0.124 (3)	−0.019 (2)	0.005 (3)	−0.003 (2)
C5	0.108 (3)	0.063 (2)	0.120 (3)	−0.018 (2)	0.017 (2)	0.002 (2)
C6	0.070 (3)	0.054 (2)	0.101 (3)	0.0013 (17)	0.012 (2)	−0.010 (2)
C7	0.065 (2)	0.053 (2)	0.107 (3)	0.0008 (17)	0.021 (2)	0.001 (2)
C8	0.057 (2)	0.047 (2)	0.094 (3)	0.0007 (17)	0.018 (2)	−0.0063 (19)
C9	0.078 (3)	0.069 (2)	0.101 (3)	−0.010 (2)	0.027 (2)	0.010 (2)
C10	0.073 (3)	0.064 (2)	0.090 (3)	−0.0117 (18)	0.014 (2)	0.002 (2)
C11	0.070 (3)	0.067 (2)	0.091 (3)	0.0036 (19)	0.025 (2)	0.009 (2)
C12	0.071 (3)	0.053 (2)	0.104 (3)	−0.0076 (18)	0.022 (2)	0.0050 (19)
C13	0.067 (3)	0.056 (2)	0.116 (3)	−0.0037 (17)	0.021 (2)	0.000 (2)
C14	0.103 (3)	0.083 (3)	0.126 (3)	−0.012 (2)	0.008 (3)	−0.008 (2)
C15	0.067 (2)	0.059 (2)	0.101 (3)	0.0070 (18)	0.022 (2)	0.001 (2)
C16	0.060 (2)	0.050 (2)	0.104 (3)	0.0043 (18)	0.019 (2)	−0.009 (2)
C17	0.072 (3)	0.058 (2)	0.106 (3)	0.0068 (19)	0.023 (2)	−0.007 (2)
C18	0.073 (3)	0.065 (2)	0.101 (3)	0.010 (2)	0.021 (2)	−0.008 (2)
C19	0.102 (4)	0.065 (3)	0.097 (4)	0.026 (2)	0.031 (3)	0.001 (2)
C20	0.090 (3)	0.069 (2)	0.111 (4)	−0.011 (2)	0.031 (3)	−0.010 (2)
C21	0.086 (3)	0.076 (3)	0.109 (4)	−0.007 (2)	0.023 (3)	−0.009 (2)
C22	0.135 (4)	0.134 (4)	0.099 (3)	0.024 (3)	0.025 (3)	−0.013 (3)

### Geometric parameters (Å, °)

**Table:**

O1—C1	1.236 (4)	C10—C11	1.363 (4)
O2—C11	1.364 (4)	C10—H10	0.9300
O2—C14	1.420 (4)	C11—C12	1.388 (4)
O3—C19	1.364 (4)	C12—C13	1.377 (4)
O3—C22	1.400 (4)	C12—H12	0.9300
C1—C6	1.477 (5)	C13—H13	0.9300
C1—C2	1.481 (5)	C14—H14A	0.9600
C2—C7	1.318 (4)	C14—H14B	0.9600
C2—C3	1.518 (4)	C14—H14C	0.9600
C3—C4	1.510 (4)	C15—C16	1.448 (4)
C3—H3A	0.9700	C15—H15	0.9300
C3—H3B	0.9700	C16—C17	1.388 (4)
C4—C5	1.498 (4)	C16—C21	1.403 (5)
C4—H4A	0.9700	C17—C18	1.364 (4)
C4—H4B	0.9700	C17—H17	0.9300
C5—C6	1.523 (4)	C18—C19	1.382 (5)
C5—H5A	0.9700	C18—H18	0.9300
C5—H5B	0.9700	C19—C20	1.372 (5)
C6—C15	1.330 (4)	C20—C21	1.352 (5)
C7—C8	1.447 (4)	C20—H20	0.9300
C7—H7	0.9300	C21—H21	0.9300
C8—C13	1.385 (4)	C22—H22A	0.9600
C8—C9	1.407 (4)	C22—H22B	0.9600
C9—C10	1.380 (4)	C22—H22C	0.9600
C9—H9	0.9300		
			
C11—O2—C14	117.8 (3)	C10—C11—C12	118.9 (4)
C19—O3—C22	120.6 (4)	O2—C11—C12	115.6 (3)
O1—C1—C6	118.9 (3)	C13—C12—C11	119.0 (3)
O1—C1—C2	120.3 (3)	C13—C12—H12	120.5
C6—C1—C2	120.7 (4)	C11—C12—H12	120.5
C7—C2—C1	118.6 (3)	C12—C13—C8	123.9 (3)
C7—C2—C3	123.8 (3)	C12—C13—H13	118.0
C1—C2—C3	117.6 (3)	C8—C13—H13	118.0
C4—C3—C2	112.9 (3)	O2—C14—H14A	109.5
C4—C3—H3A	109.0	O2—C14—H14B	109.5
C2—C3—H3A	109.0	H14A—C14—H14B	109.5
C4—C3—H3B	109.0	O2—C14—H14C	109.5
C2—C3—H3B	109.0	H14A—C14—H14C	109.5
H3A—C3—H3B	107.8	H14B—C14—H14C	109.5
C5—C4—C3	111.6 (3)	C6—C15—C16	133.6 (3)
C5—C4—H4A	109.3	C6—C15—H15	113.2
C3—C4—H4A	109.3	C16—C15—H15	113.2
C5—C4—H4B	109.3	C17—C16—C21	114.5 (4)
C3—C4—H4B	109.3	C17—C16—C15	120.4 (4)
H4A—C4—H4B	108.0	C21—C16—C15	125.0 (4)
C4—C5—C6	110.7 (3)	C18—C17—C16	124.9 (4)
C4—C5—H5A	109.5	C18—C17—H17	117.5
C6—C5—H5A	109.5	C16—C17—H17	117.5
C4—C5—H5B	109.5	C17—C18—C19	118.1 (4)
C6—C5—H5B	109.5	C17—C18—H18	121.0
H5A—C5—H5B	108.1	C19—C18—H18	121.0
C15—C6—C1	119.3 (3)	O3—C19—C20	117.0 (4)
C15—C6—C5	122.6 (3)	O3—C19—C18	124.0 (4)
C1—C6—C5	118.1 (3)	C20—C19—C18	118.9 (4)
C2—C7—C8	136.0 (3)	C21—C20—C19	121.9 (4)
C2—C7—H7	112.0	C21—C20—H20	119.0
C8—C7—H7	112.0	C19—C20—H20	119.0
C13—C8—C9	115.2 (3)	C20—C21—C16	121.5 (4)
C13—C8—C7	119.9 (3)	C20—C21—H21	119.3
C9—C8—C7	124.8 (3)	C16—C21—H21	119.3
C10—C9—C8	121.3 (3)	O3—C22—H22A	109.5
C10—C9—H9	119.3	O3—C22—H22B	109.5
C8—C9—H9	119.3	H22A—C22—H22B	109.5
C11—C10—C9	121.6 (3)	O3—C22—H22C	109.5
C11—C10—H10	119.2	H22A—C22—H22C	109.5
C9—C10—H10	119.2	H22B—C22—H22C	109.5
C10—C11—O2	125.5 (3)		

### Hydrogen-bond geometry (Å, °)

**Table:**

*D*—H···*A*	*D*—H	H···*A*	*D*···*A*	*D*—H···*A*
C14—H14B···O2^i^	0.96	2.67	3.512 (5)	146
C4—H4A···O1^ii^	0.97	2.61	3.510 (5)	154

Symmetry codes: (i) −*x*+1, −*y*, −*z*+1; (ii) −*x*, −*y*, −*z*.
